# Major adverse cardiac events and mortality in chronic obstructive pulmonary disease following percutaneous coronary intervention: a systematic review and meta-analysis

**DOI:** 10.1186/s12872-017-0622-2

**Published:** 2017-07-17

**Authors:** Pravesh Kumar Bundhun, Chakshu Gupta, Guang Ma Xu

**Affiliations:** 1 Institute of Cardiovascular Diseases, the First Affiliated Hospital of Guangxi Medical University, Nanning, Guangxi 530027 People’s Republic of China; 2 Guangxi Medical University, Nanning, Guangxi 530027 People’s Republic of China; 3 Department of Cardiology, The People’s Hospital of Guangxi Zhuang Autonomous Region, Nanning, Guangxi 530021 People’s Republic of China

**Keywords:** Chronic obstructive pulmonary diseases, Percutaneous coronary intervention, Mortality, Major adverse cardiac events

## Abstract

**Background:**

We aimed to systematically compare Major Adverse Cardiac Events (MACEs) and mortality following Percutaneous Coronary Intervention (PCI) in patients with and without Chronic Obstructive Pulmonary Diseases (COPD) through a meta-analysis.

**Methods:**

Electronic databases (Cochrane library, EMBASE and Medline/PubMed) were searched for English publications comparing in-hospital and long-term MACEs and mortality following PCI in patients with a past medical history of COPD. Statistical analysis was carried out by Revman 5.3 whereby Odds Ratio (OR) and 95% Confidence Intervals (CI) were considered the relevant parameters.

**Results:**

A total number of 72,969 patients were included (7518 patients with COPD and 65,451 patients without COPD). Results of this analysis showed that in-hospital MACEs were significantly higher in the COPD group with OR: 1.40, 95% CI: 1.19–1.65; *P* = 0.0001, I^2^ = 0%. Long-term MACEs were still significantly higher in the COPD group with OR: 1.58, 95% CI: 1.38–1.81; *P* = 0.00001, I^2^ = 29%. Similarly, in-hospital and long-term mortality were significantly higher in patients with COPD, with OR: 2.25, 95% CI: 1.78–2.85; *P* = 0.00001, I^2^ = 0% and OR: 2.22, 95% CI: 1.33–3.71; *P* = 0.002, I^2^ = 97% respectively. However, the result for the long-term death was highly heterogeneous.

**Conclusion:**

Since in-hospital and long-term MACEs and mortality were significantly higher following PCI in patients with versus without COPD, COPD should be considered a risk factor for the development of adverse clinical outcomes following PCI. However, the result for the long-term mortality was highly heterogeneous warranting further analysis.

## Background

In this new era of 2016–2017, where the total number of smokers has increased drastically among the youngsters and the older population of males and females (smoking cigarettes began at a very young age, female smokers are on the rise, and passive smokers are becoming more and more common), Chronic Obstructive Pulmonary Diseases (COPD) might soon overcome other major worldwide causes of death [1, 2]. Even though this severe chronic respiratory disease is known from decades, limited data are available on patients who suffer coronary co-morbidities and who are candidates for Percutaneous Coronary Intervention (PCI).

Among the few researches which were undertaken, controversies have already been observed among COPD patients who underwent PCI. Insights from the National Heart, Lung and Blood Institute Dynamic Registry showed COPD to be associated with higher Major Adverse Cardiac Events (MACEs) and mortality rates following PCI [3]. In contrast, in a research which was carried out in a hospital in Taiwan, the authors concluded that COPD was not an independent predictor of major adverse clinical outcomes in patients with STEMI following PCI [4]. This same study also unexpectedly showed no difference in hospital mortality between COPD and non-COPD patients following PCI.

Therefore, we aimed to systematically compare MACEs and mortality following PCI, in patients with and without COPD, through a meta-analysis.

## Methods

### Data sources and search strategies

Electronic databases (Cochrane library, EMBASE and Medline/PubMed) were searched for English publications related to COPD and PCI by typing the following words or phrases each at a time:

- Chronic obstructive pulmonary disease and percutaneous coronary intervention
- COPD and PCI
- COPD and coronary angioplasty
- COPD and myocardial infarction

If an article appeared to be fully relevant, its reference list was also checked for any suitable study.

### Inclusion criteria

Studies which satisfied the inclusion criteria were those studies that:

- Were randomized trials or observational studies which compared PCI in patients with versus without COPD.
- Reported either MACEs or death among their clinical outcomes.
- Involved data which were relevant to this current analysis.

### Exclusion criteria

Studies were excluded if:

- They were other types of studies apart from randomized trials or observational studies.
- They did not involve patients with COPD.
- They did not report either MACEs or mortality among their clinical endpoints.
- They were either duplicates or involved the same cohort or trial.

### Definitions, outcomes and follow ups

COPD was defined differently in different studies. COPD, as defined in each of the study has been listed in Table 1.

**Table 1:** Definitions of chronic obstructive pulmonary disease within the different studies

Studies	Definitions
Almagro 2015	COPD was defined as a post-bronchodilator forced expiratory volume in the 1st second (FEV1)/forced vital capacity (FVC) ratio < 0.70.
Berger 2004	COPD was defined by the requirement of chronic bronchodilator therapy or a forced expiratory volume in 1 s < 75% of the predicted value or a room air pO2 < 60 or a pCO2 > 50.
Campo 2013	A patient was considered to have COPD combining different sources of data: i) documented history of hospital admission for COPD; ii) treatment with pharmacologic therapies specific for COPD (e.g., inhaled steroids, inhaled anticholinergics, inhaled β-agonists or theophylline).
Enriquez 2011	COPD was defined as a history or presence of physician-diagnosed COPD. Additionally, the patients were required to be on chronic pharmacologic therapy and/or have an FEV_1_ < 75% of predicted value.
Jatene 2016	The presence of COPD was determined clinically by local investigators, based on history, clinical presentation, previous examinations, and medications, recorded as COPD in the case report form at enrollment.
Konecny 2010	Very severe COPD was defined as an FEV 1 /FVC ratio ≤ 70% and an FEV 1 ≤ 30% predicted, severe COPD as an FEV 1/FVC ratio ≤ 70% and an FEV 1 between 30% and 50% predicted, and mild-to-moderate COPD as an FEV 1/FVC ratio ≤ 70 and an FEV 1 > 50% predicted.
Nishiyama 2009	A patient was considered to have COPD if it was listed as a comorbid condition in our database and its diagnosis was confirmed by a simple test called spirometry. Such a diagnosis should be considered in any patient who has symptoms of cough, sputum production, or dyspnea (difficult or labored breathing), and/or a history of exposure to risk factors for the disease. In cases where spirometry is unavailable, the diagnosis of COPD should be made using all available tools. Clinical symptoms and signs such as abnormal shortness of breath and increased forced expiratory time can be used to arrive at the diagnosis.
Selvaraj 2005	The diagnosis of COPD was based on the clinical history or obtained from chart review and recorded as a co-morbidity in the database.
Sung 2013	COPD was defined according to one of the following criteria: (1) Information on COPD status was obtained by reviewing chart record of the need for pharmacologic therapy using bronchodilator agent; (2) Past history of a 1-s forced expiratory volume < 70% of the predicted value (by pulmonary function test); (3) Physical examination (by auscultation) showed expiratory wheezing and further confirmed by blood gas and chest radiograph (i.e., emphysematous change); or (4) Current use of bronchodilators prior to acute myocardial infarction.
Zhang 2012	A diagnosis of COPD should be considered in any patient who has symptoms of cough, sputum production, or dyspnea, and/or a history of exposure to risk factors for the disease. The diagnosis is confirmed by spirometry. The presence of a postbronchodilator FEV1 < 80% of the predicted value in combination with an FEV1/FVC < 70% confirms the presence of airflow limitation that is not fully reversible. Where spirometry is unavailable, the diagnosis of COPD should be made using all available tools.

*Abbreviations*: *COPD* chronic obstructive pulmonary disease, *FEV* forced expiratory volume, *FVC* forced vital capacity

The main outcomes which were analyzed included:

- MACEs which consisted of death, myocardial infarction (MI), repeated revascularization or another clinical outcome.
- Mortality (all-cause death)
- MI
- Coronary revascularization (CR)

Follow up periods included:

- In-hospital follow up
- A longer follow up period greater than one year.

The definitions of the outcomes were listed in Table 2 and the reported outcomes and follow up periods were summarized in Table 3.

**Table 2:** Definition of outcomes and follow-up periods

Outcomes	Definitions
Major adverse cardiac events (MACEs)	Defined as a combination of several outcomes including death, MI and revascularization
Death	Defined as all-cause mortality, that is, mortality due to any medical reason including cardiac and non-cardiac
Myocardial infarction (MI)	Defined as re-infarction that occurred post percutaneous coronary intervention based on two or more of the following: 1. Typical chest pain, 2. ECG showing ST-T or Q wave changes, 3. Increase in serum enzyme (creatinine kinase, lactate dehydrogenase or troponin), 4. New wall motion abnormalities on ultrasound
Coronary revascularization (CR)	Defined as repeated revascularization in the coronary arteries resulting in re-stenosis
In-hospital follow-up	Defined as the follow-up period during their hospital stay (≤ 1 month)
Long-term follow-up	Defined as the follow-up period of one or more years

*Abbreviations*: *ECG* electrocardiogram

**Table 3:** Reported outcomes and follow up periods

Studies	Outcomes	Follow up period
Almagro 2015 [8]	Death	3 years
Berger 2004 [9]	MACEs, MI	In-hospital
Campo 2013 [10]	Death, MI, CR	In-hospital and 3 years
Enriquez 2011 [3]	Death, MI, MACEs, CR	In-hospital and 1 year
Jatene 2016 [11]	Death, MACEs, MI, CR	2 years
Konecny 2010 [12]	Death, MI	10 years
Nishiyama 2009 [13]	Death, MACEs, MI	In-hospital, 1–4 years
Selvaraj 2005 [14]	Death, MI	In-hospital
Sung 2013 [4]	MACEs	1 year
Zhang 2012 [15]	Death, MI, MACEs, CR	In-hospital

*Abbreviations*: *MACEs* major adverse cardiac events, *MI* myocardial infarction, *CR* coronary revascularization

### Data extraction and review

The following data were extracted by two independent reviewers (PKB and CG):

- Author names;
- Publication year;
- Types of study;
- Year of patients’ enrollment;
- Number of patients with COPD;
- Number of patients without COPD;
- Outcomes reported in each study;
- The follow up periods;
- The baseline characteristics of the patients (those with and without COPD) including the mean age, percentage of male patients, percentage of patients suffering from hypertension, dyslipidemia, diabetes mellitus and current smokers;
- Number of events in the study (COPD) as well as the control (non-COPD) groups.

Any disagreement which followed were discussed carefully with each other. However, any unsolved issue was further discussed by the third author (GMX) and a final decision was made by him. In this meta-analysis, the PRISMA guideline was followed [5].

The Newcastle Ottawa Scale (NOS) was used to assess the methodological quality of the studies (non-randomized studies) and NOS has been refined based on expertise and experience whereby it was used in several projects [6].

This NOS consists of eight items, which have been categorized in three different groups: selection, comparability, and outcome or exposure. These three groups involved several sub-items whereby ‘stars’ were given if these items were present. Each item deserved one star, however, a maximum of 2 stars could be given for comparability.

Selection included (maximum 4 stars): representativeness of the exposed cohort, selection of the non-exposed cohort, ascertainment of exposure, demonstrating the fact that any outcome of interest was not present at the beginning of the study.

Comparability included (maximum 2 stars): comparability of the cohorts on the basis of the design or analysis.

Outcome included (maximum 3 stars): assessment of the outcome, longer duration of the follow up, adequacy of follow-up of cohorts.

NOS assessment involved a minimum number of zero star to a maximum number of nine stars depending on the quality of the study being assessed. The total number of scores allotted were listed in Table 4.

**Table 4:** Study assessment using the Newcastle Ottawa Scale

Studies	Stars allocated following NOS assessment	No of stars (n)
Almagro 2015	*******	7
Berger 2004	******	6
Campo 2013	********	8
Enriquez 2011	*******	7
Konecny 2010	********	8
Nishiyama 2009	*******	7
Selvaraj 2005	******	6
Sung 2013	******	6
Zhang 2012	******	6

*Abbreviations*: *NOS* Newcastle Ottawa scale

### Statistical analysis

Statistical analysis was carried out by the latest version of Revman software (5.3) whereby odds ratio (OR) and 95% confidence intervals (CI) were considered relevant.

During the subgroup analysis, heterogeneity [7] was assessed by the Q statistic test focusing on the *P* value with a cut-off point of 0.05. A *P* value less or equal to 0.05 was considered statistically significant or else, the result was considered insignificant.

Heterogeneity was also dependent on the I^2^ test. A low heterogeneity was denoted by a low percentage of I^2^ whereas an increasing percentage denoted an increasing heterogeneity.

The decision to use a fixed effects model (I^2^ < 50%) or a random effects model (I^2^ > 50%) was also dependent upon the I^2^ value.

Publication bias was visually estimated through funnel plots.

Sensitivity analyses were also carried out by the exclusion method (each study was excluded one by one and a new analysis was carried out each time).

Ethical Board Review approval was not required.

## Results

### Flow of study selection

**Fig. 1:**
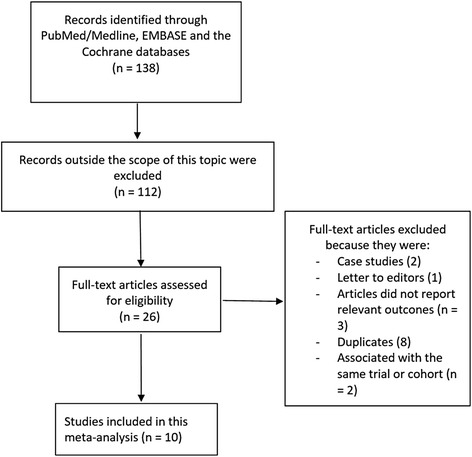
Flow diagram representing the study selection

Figure 1 represents the process of the study selection. In all, a total number of 138 publications was obtained through the electronic search. After a careful assessment of the titles and a close check of the abstracts, 112 articles were eliminated (not related to the idea of this research). Twenty-six full text articles were assessed for eligibility. Further articles were deselected since they were either case studies (2), letter to editors (1), they did not report the relevant endpoints (3), they were duplicates (8) or they were associated with the same trial or cohort (2). Finally, 10 studies [3, 4, 8–15] were selected for this analysis.

### General features of the studies which were included

A total number of 72,969 patients were included (7518 patients with COPD and 65,451 patients without COPD) in this analysis. Most of the studies were observational studies and the enrollment period of the patients ranged from 1997 to 2011 (Table 5).

**Table 5:** General features of the studies which were included

Studies	Patients enrollment period	Types of study	No of patients with COPD (n)	No of patients without COPD (n)	Total no of patients (n)
Almagro 2015	2011	Observational	33	100	133
Berger 2004	1998–1999	Observational	183	4101	4284
Campo 2013	2003–2009	Observational	2032	9086	11,118
Enriquez 2011	1997–2006	Observational	860	10,048	10,908
Jatene 2016	-	RCT	283	4322	4605
Konecny 2010	2005–2008	Observational	2001	12,345	14,346
Nishiyama 2009	2000–2002	Observational	240	9632	9872
Selvaraj 2005	1997–2003	Observational	1117	9877	10,994
Sung 2013	2002–2011	Observational	124	1430	1554
Zhang 2012	2006–2011	Observational	645	4510	5155
Total (*n*)			7518	65,451	72,969

*Abbreviations*: *COPD*chronic obstructive pulmonary disease, *RCT*randomized controlled trial

### Baseline features of the studies which were included

Baseline features have been summarized in Table 6. A mean age ranging from 66.1 to 70.0 in the COPD group and 60.9 to 66.0 in the non-COPD group were observed. Most of the patients were males with a percentage above 50% in each study. The percentage of patients with co-morbidities such as hypertension, dyslipidemia, and diabetes mellitus has been listed in Table 6. Current smokers were slightly higher in the COPD group compared to the non-COPD group. Overall, almost no significant difference was observed in the baseline features of the patients in both of the groups.

**Table 6:** Baseline features of the patients

Studies	Age (yrs)	Males (%)	Ht (%)	Ds (%)	Cs (%)	DM (%)
	**+/−**	**+/−**	**+/−**	**+/−**	**+/−**	**+/−**
Almagro 2015	67.5/61.6	84.8/76.0	69.7/69.0	66.7/62.0	18.2/17.0	36.4/27.0
Berger 2004	66.1/63.3	56.0/69.0	71.0/70.0	−	30.0/22.0	30.0/27.0
Campo 2013	70.0/65.0	66.0/74.0	70.0/61.0	46.8/48.3	24.0/27.0	21.8/20.9
Enriquez 2011	66.8/63.2	57.0/66.1	78.1/69.7	67.0/70.0	30.9/24.4	36.9/30.2
Jatene 2016	67.8/63.0	75.6/76.7	74.6/63.6	67.8/63.0	42.4/33.8	24.4/16.8
Konecny 2010	69.9/66.0	72.0/70.0	74.0/70.0	73.0/76.0	30.0/17.0	26.0/24.0
Nishiyama 2009	−	82.5/70.4	62.5/69.2	−	43.8/35.7	31.7/39.0
Selvaraj 2005	67.6/64.1	62.0/71.1	75.1/71.6	17.2/20.1	27.0/18.0	37.2/30.5
Sung 2013	68.5/60.9	85.5/81.2	54.8/55.6	39.5/42.3	37.1/34.5	32.3/36.2
Zhang 2012	68.4/64.7	73.0/71.0	75.0/71.0	65.0/63.0	38.0/29.0	25.0/22.0

*Abbreviations*: *yrs.* years, *Ht* hypertension, *Ds* dyslipidemia, *Cs* current smoking, *DM*diabetes mellitus, ‘+’: *COPD* ‘-’: no COPD

### MACEs following PCI in patients with COPD versus patients without COPD

**Fig. 2:**
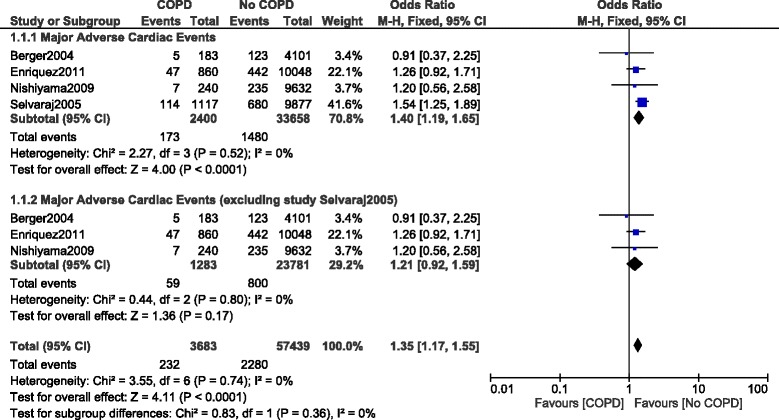
In-hospital major adverse cardiac events reported in patients with versus without COPD

Results of this analysis showed that in-hospital MACEs were significantly higher in the COPD group with OR: 1.40, 95% CI: 1.19–1.65; *P* = 0.0001, I^2^ = 0%. It was also observed that this result might have been influenced by the study Selvaraj 2005. Therefore, another analysis was carried out with the exclusion of study Selvaraj 2005. This time, even if in hospital MACEs were higher in the COPD group with OR: 1.21, 95% CI: 0.92–1.59; *P* = 0.17, I^2^ = 0%, the result was not statistically significant. Results illustrating in-hospital MACEs have been represented in Fig. 2.

**Fig. 3:**
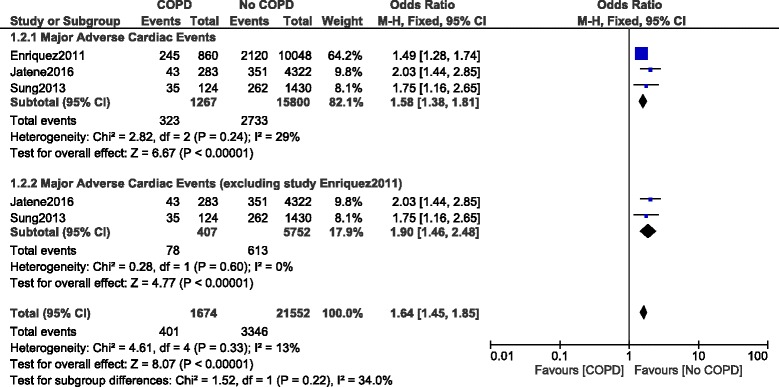
Long-term major adverse cardiac events reported in patients with versus without COPD

When MACEs were analyzed during a longer follow up period, MACEs were significantly higher in the COPD group with OR: 1.58, 95% CI: 1.38–1.81; *P* = 0.00001, I^2^ = 29%. This time, it came to our attention that the result might have been influenced by study Enriquez 2011. Therefore, when study Enriquez 2011 was excluded and another analysis was conducted, MACEs still significantly favored non-COPD with OR: 1.90, 95% CI: 1.46–2.48; *P* = 0.00001, I^2^ = 0% during this longer follow up period. Results showing MACEs during the longer follow up have been illustrated in Fig. 3.

### Mortality following PCI in COPD versus non-COPD

**Fig. 4:**
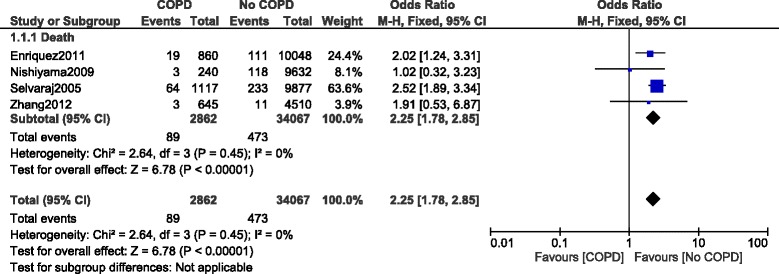
In-hospital mortality reported in patients with versus without COPD

When mortality was analyzed, in-hospital death was significantly higher in patients with COPD, with OR: 2.25, 95% CI: 1.78–2.85; *P* = 0.00001, I^2^ = 0% (Fig. 4).

**Fig. 5:**
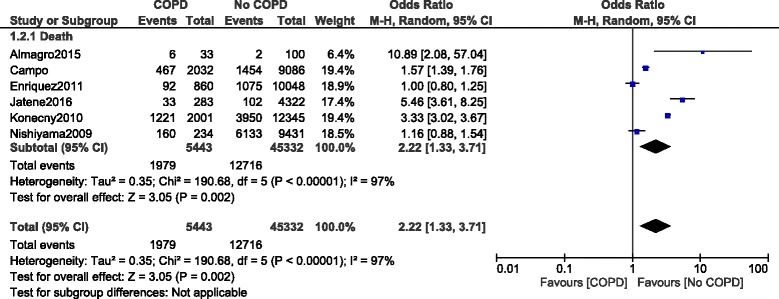
Long-term mortality reported in patients with versus without COPD

During a longer follow up period, mortality was still significantly higher in the COPD group with OR: 2.22, 95% CI: 1.33–3.71; *P* = 0.002, I^2^ = 97%. However, even if it was fully relevant to the literature, this long-term result was highly heterogeneous (Fig. 5).

### Other outcomes following PCI in patients with versus without COPD

**Fig. 6:**
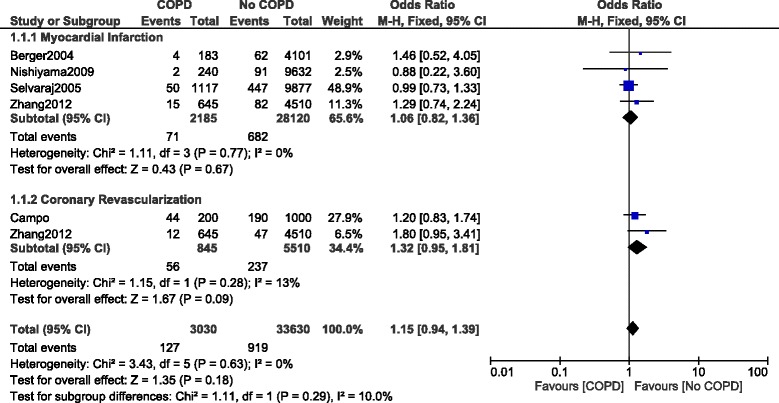
Other in-hospital outcomes reported in patients with versus without COPD

Other clinical outcomes were also analyzed. Our results showed that in-hospital MI and CR were not significantly different with OR: 1.06, 95% CI: 0.82–1.36; *P* = 0.67, I^2^ = 0% and OR: 1.32, 95% CI: 0.95–1.81; *P* = 0.09, I^2^ = 13% respectively (Fig. 6).

**Fig. 7:**
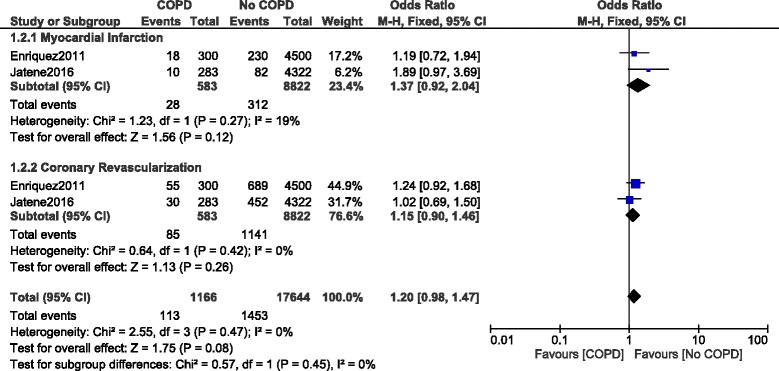
Other long-term outcomes reported in patients with versus without COPD

When a longer follow up was considered, MI and CR were still not significantly different with OR: 1.37, 95% CI: 0.92–2.04; *P* = 0.12, I^2^ = 19% and OR: 1.15, 95% CI: 0.90–1.46; *P* = 0.26, I^2^ = 0 respectively (Fig. 7).

### Analysis including patients with COPD which was confirmed by a spirometry test

**Fig. 8:**
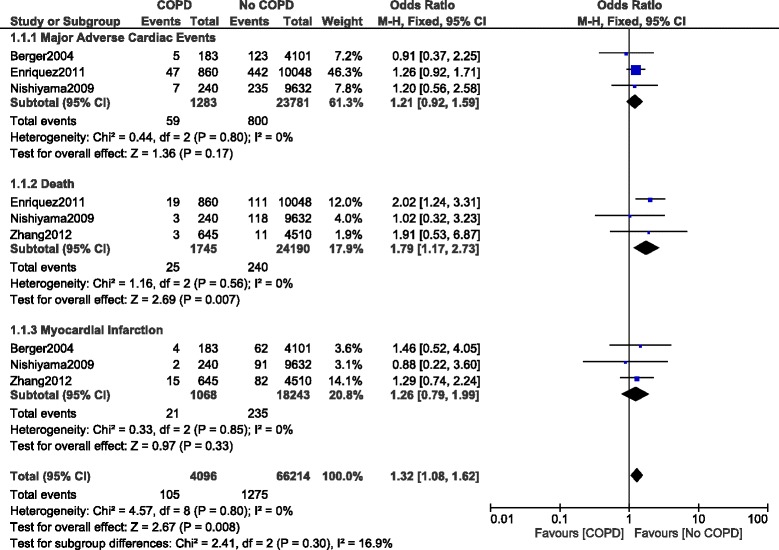
In-hospital outcomes reported in patients with versus without COPD (defined with respect to the spirometry test)

Another subgroup analysis was carried out including patients with COPD defined based on a spirometry test. In-hospital mortality was still significantly higher in patients with COPD with OR: 1.79, 95% CI: 1.17–2.73; *P* = 0.007, I^2^ = 0%. However, MACEs and MI were not significantly different with OR: 1.21, 95% CI: 0.92–1.59; *P* = 0.17, I^2^ = 0% and OR: 1.26, 95% CI: 0.79–1.99; *P* = 0.33, I^2^ = 0% respectively as shown in Fig. 8.

**Fig. 9:**
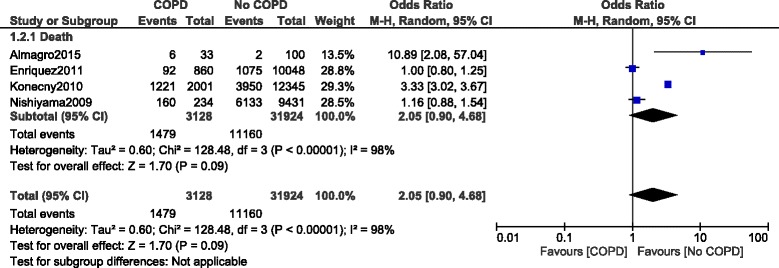
Long-term mortality reported in patients with versus without COPD (defined with respect to the spirometry test)

In addition, long-term death in this particular subgroup of patients was not significantly different with OR: 2.05, 95% CI: 0.90–4.68; *P* = 0.09, I^2^ = 98% as shown in Fig. 9.

Sensitivity analyses obtained consistent results. Table 7 summarized the overall results of this analysis.

**Table 7:** Results of this analysis

Outcomes analyzed	No of studies included	OR with 95% CI	*P* value	I^2^ (%)
In-hospital follow up				
MACEs	4	1.40 [1.19–1.65]	0.0001	0
MACEs	3	1.21 [0.92–1.59]	0.17	0
Death	4	2.25 [1.78–2.85]	0.00001	0
MI	4	1.06 [0.82–1.36]	0.67	0
CR	2	1.32 [0.95–1.81]	0.09	13
Above 1 year follow up				
MACEs	3	1.58 [1.38–1.81]	0.00001	29
MACEs	2	1.90 [1.46–2.48]	0.00001	0
Death	6	2.22 [1.33–3.71]	0.002	97
MI	2	1.37 [0.92–2.04]	0.12	19
CR	2	1.15 [0.90–1.46]	0.26	0

*Abbreviations*: *MACEs* major adverse cardiac events, *MI* myocardial infarction, *CR* coronary revascularization, *OR* odds ratio, *CI* confidence intervals

### Publication bias

**Fig. 10:**
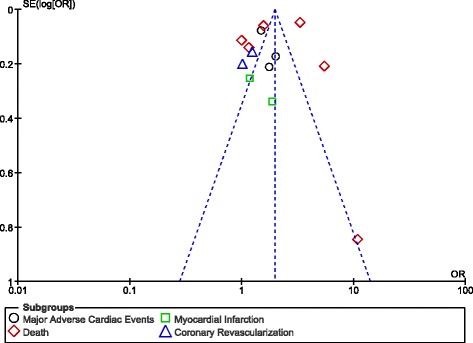
Funnel plot showing publication bias

**Fig. 11:**
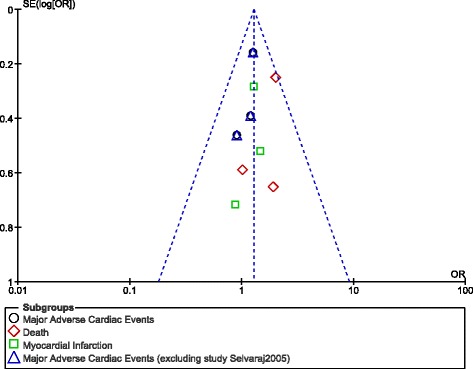
Funnel plot showing publication bias

Based on the funnel plots obtained (Figs. 10 and 11), there was only little evidence of publication bias among the studies which assessed most of the clinical endpoints.

## Discussion

Since the total number of patients with COPD is on the rise, this analysis aimed to compare MACEs and mortality following PCI in patients with and without COPD. Results of this analysis showed in-hospital as well as long-term MACEs to be higher in COPD patients following coronary angioplasty. Mortality was also significantly higher during the in-hospital and longer follow-up periods. However, other clinical outcomes which were analyzed (MI and CR) were not significantly different between these 2 groups of patients.

Several studies have shown an association of cardiovascular diseases with COPD. Cardiovascular diseases accounts for a high portion of mortality in such chronic pulmonary patients. A recent systematic review summarizing the existing data regarding subclinical cardiovascular events in patients with COPD on the base of identifying screening strategies in such patients showed a high subclinical burden of coronary artery disease in these chronic pulmonary patients [16]. Other studies have shown carotid-intima media thickness to significantly increase in patients with COPD suffering from coronary artery disease [17]. Recent research has also shown COPD to also be very prevalent in European patients with atrial fibrillation, and these patients were at a higher risk of several cardiovascular complications and death [18].

Well, to support the results of this current analysis, a study involving 1 of 3 tertiary medical centers in New York City showed COPD to be independently associated with long-term mortality following PCI [9]. Another study published by Selvaraj et al. and including 10,994 patients also showed a higher in-hospital and long-term mortality to be associated with COPD [14]. In addition, Insights from the National Heart, Lung and Blood Institute Dynamic Registry also showed worse prognosis in patients with COPD following PCI [3]. The authors even concluded that a lower rate of guidelines recommended class I medications which were prescribed at discharge might be hugely responsible for such higher death rates. Our results were further supported by the REAL registry [10].

Nevertheless, a few studies did not report significantly high post-angioplasty MACEs or mortality associated with COPD. The study published by Sung et al. which aimed to report the incidence and prognostic outcome in COPD patients with acute coronary syndrome (NSTEMI) showed COPD not to be an independent predictor of short and medium-term major adverse clinical outcomes in such patients following PCI [4].

Finally, according to our observations, it is recommended that special care and strict medical adherence have to be considered when managing COPD patients following PCI to avoid or reduce re-admission to the hospital, exacerbation of the obstructive disease, or any increase in mortality or MACEs following PCI. An overview of the pharmacological challenges facing physicians in the management of patients with concomitant cardiovascular disease and COPD strongly suggests that evidence-based treatment in such cases should not be changed [19]. In daily practice in clinics, obtaining the optimal titration of cardiovascular and respiratory drugs is a vital element. Early identification of co-morbidities and counselling about the harm of cigarette smoking might help to improve prognosis in such patients.

### Novelty

This research contributes to a novel aspect in clinical medicine due to the fact that it is the first meta-analysis of COPD versus non-COPD and PCI. The larger population size might also contribute to its novelty. In addition, a low level of heterogeneity which was obtained among several subgroups which were analyzed could represent another new feature of this analysis.

### Limitations

Limitations of this research were:

- Even though a large number of patients was used, this number might still be small compared to other studies outside this scope.
- Most of the studies which were included were observational studies with heterogeneous data. Therefore, the subgroup analyzing long-term mortality involved a very high level of heterogeneity.
- In addition, different studies had different follow-up periods further contributing to this high level of heterogeneity when analyzing long-term mortality.
- When other clinical outcomes (MI and CR) were analyzed, in some cases, the number of patients were adjusted to avoid the influence of studies with larger number of patients. This might also have affect the results for other clinical outcomes.
- Important endpoints were not reported in all the studies. A few studies reported MACEs while others reported mortality. Therefore, only a few studies were available for comparison during the subgroup analysis.

## Conclusion

Since in-hospital and long-term MACEs and mortality were significantly higher following PCI in patients with versus without COPD, COPD should be considered a risk factor for the development of adverse clinical outcomes following PCI. However, the result for the long-term mortality was highly heterogeneous warranting further analysis.

## Abbreviations

COPD: Chronic obstructive pulmonary disease
MACEs: Major adverse cardiac events
PCI: Percutaneous coronary intervention
